# The impact of COVID-19 critical illness on new disability, functional outcomes and return to work at 6 months: a prospective cohort study

**DOI:** 10.1186/s13054-021-03794-0

**Published:** 2021-11-08

**Authors:** Carol L. Hodgson, Alisa M. Higgins, Michael J. Bailey, Anne M. Mather, Lisa Beach, Rinaldo Bellomo, Bernie Bissett, Ianthe J. Boden, Scott Bradley, Aidan Burrell, D. James Cooper, Bentley J. Fulcher, Kimberley J. Haines, Jack Hopkins, Alice Y. M. Jones, Stuart Lane, Drew Lawrence, Lisa van der Lee, Jennifer Liacos, Natalie J. Linke, Lonni Marques Gomes, Marc Nickels, George Ntoumenopoulos, Paul S. Myles, Shane Patman, Michelle Paton, Gemma Pound, Sumeet Rai, Alana Rix, Thomas C. Rollinson, Janani Sivasuthan, Claire J. Tipping, Peter Thomas, Tony Trapani, Andrew A. Udy, Christina Whitehead, Isabelle T. Hodgson, Shannah Anderson, Ary Serpa Neto, The COVID-Recovery Study Investigators and the ANZICS Clinical Trials Group, Nicola Burgess, Kirsty Hearn, David Brewster, Alyssa Waanders, Shannon Simpson, Yasmin de Silva, Jenna Lang, Sarah Burleigh, Elisha Killer, Michael Wang, Lauren O’Connor, Lauren Thomas, Lucy Dennis, Joanna Caruana, Wisam Al-Bassam, Morag Shealy, Marianne Chapman, Stephanie O’Connor, Janne Sheehan, Emily Alexander, Amanda Sukkar, Liesl Davis, Francis Bass, Naomi Hammond, Anne O’Connor, Elizabeth Yarad, Richard Totaro Heidi Buhr, Nazmeen Reddy, Wendy Chaseling, Kelvin Ip, Oystein Tronstad, Alison Mahoney, Cadi Fanning, Hariette Esterman, Alexia Kozary, Bronte Scott, Donna Urquhart

**Affiliations:** 1 Australian and New Zealand Intensive Care Research Centre, School of Public Health and Preventive Medicine, Monash University, Melbourne, Victoria Australia; 2 Department of Intensive Care and Hyperbaric Medicine, The Alfred, Melbourne, Victoria Australia; 3 Department of Physiotherapy, The Alfred, Melbourne, Victoria Australia; 4 Department of Critical Care, School of Medicine, University of Melbourne, Victoria, Australia; 5 Department of Physiotherapy (Allied Health), The Royal Melbourne Hospital, Melbourne, Victoria Australia; 6 Discipline of Physiotherapy, University of Canberra, Canberra, Australia; 7 Physiotherapy Department, Canberra Hospital, Canberra, Australia; 8 Physiotherapy Department, Launceston General Hospital, Launceston, Tasmania Australia; 9 Launceston Clinical School, University of Tasmania, Tasmania, Australia; 10 Physiotherapy Department, Western Health, Melbourne, Victoria Australia; 11 School of Health and Rehabilitation Sciences, University of Queensland, Brisbane, Queensland Australia; 12 Intensive Care Medicine Nepean Hospital, New South Wales, Australia; 13 Fiona Stanley Hospital, Perth, Western Australia Australia; 14 Physiotherapy Department, Princess Alexandra Hospital, Metro South Health, Queensland, Australia; 15 Physiotherapy, St Vincent’s Hospital, Sydney, New South Wales Australia; 16 Department of Anaesthesiology and Perioperative Medicine, Central Clinical School, Monash University, Melbourne, Victoria Australia; 17 Faculty of Medicine, Nursing and Midwifery, Health Sciences and Physiotherapy, The University of Notre Dame Australia, Perth, Western Australia Australia; 18 Department of Physiotherapy, Monash Health, Melbourne, Victoria Australia; 19 Physiotherapy Department, St Vincent’s Hospital, Melbourne, Victoria Australia; 20 Canberra Health Services, Canberra, Australia; 21 Medical School, Australia National University, Canberra, Australia; 22 Department of Physiotherapy, Division of Allied Health, Austin Health, Melbourne, Australia; 23 Department of Physiotherapy, The University of Melbourne, Melbourne, Victoria Australia; 24 Department of Physiotherapy, Royal Brisbane and Women’s Hospital, Brisbane, Queensland Australia; 25 Data Analytics Research and Evaluation (DARE) Centre, Austin Hospital, Melbourne, Victoria Australia; 26 Department of Critical Care Medicine, Hospital Israelita Albert Einstein, Sao Paulo, Brazil

**Keywords:** Intensive care, Disability, COVID-19, Mechanical ventilation, Long-term outcome

## Abstract

**Background:**

There are few reports of new functional impairment following critical illness from COVID-19. We aimed to describe the incidence of death or new disability, functional impairment and changes in health-related quality of life of patients after COVID-19 critical illness at 6 months.

**Methods:**

In a nationally representative, multicenter, prospective cohort study of COVID-19 critical illness, we determined the prevalence of death or new disability at 6 months, the primary outcome. We measured mortality, new disability and return to work with changes in the World Health Organization Disability Assessment Schedule 2.0 12L (WHODAS) and health status with the EQ5D-5L^TM^.

**Results:**

Of 274 eligible patients, 212 were enrolled from 30 hospitals. The median age was 61 (51–70) years, and 124 (58.5%) patients were male. At 6 months, 43/160 (26.9%) patients died and 42/108 (38.9%) responding survivors reported new disability. Compared to pre-illness, the WHODAS percentage score worsened (mean difference (MD), 10.40% [95% CI 7.06–13.77]; *p* < 0.001). Thirteen (11.4%) survivors had not returned to work due to poor health. There was a decrease in the EQ-5D-5L^TM^ utility score (MD, − 0.19 [− 0.28 to − 0.10]; *p* < 0.001). At 6 months, 82 of 115 (71.3%) patients reported persistent symptoms. The independent predictors of death or new disability were higher severity of illness and increased frailty.

**Conclusions:**

At six months after COVID-19 critical illness, death and new disability was substantial. Over a third of survivors had new disability, which was widespread across all areas of functioning.

*Clinical trial registration* NCT04401254 May 26, 2020.

**Supplementary Information:**

The online version contains supplementary material available at 10.1186/s13054-021-03794-0.

## Background

The coronavirus disease 2019 (COVID-19) pandemic has had a tremendous impact on global health [1]. The disease spectrum of severe acute respiratory syndrome coronavirus type 2 (SARS-CoV-2) infection is wide, ranging from asymptomatic to critical illness [2, 3]. Because COVID-19 is a new disease, the impact on long-term outcomes in survivors is still emerging. Similar to other post viral syndromes, there are reports of prolonged effects after acute COVID-19 [3–8]. Survivors have contributed to the recognition of a syndrome called “Long COVID,” characterized by persistent symptoms or long-term complications, but there is no agreed clinical definition of Long COVID, nor a clear treatment pathway [8–10].

The critically ill population is likely to be especially vulnerable to the prolonged effects of COVID-19, but it is possible that the effects of COVID-19 are similar to other illnesses. Survivors of critical illnesses have previously reported long-term impairments in physical, cognitive and/or psychological function, often known as post-intensive care syndrome [11, 12]. Study of the sequelae in survivors of COVID-19 critical illness is urgently needed. This will allow clinicians to develop an evidence-based multidisciplinary approach for management of these patients, and to inform research priorities [13, 14]. While there have been several reports about ongoing symptoms, the impact of these on new disability remains unclear. The aim of this study was to describe the incidence of a poor outcome defined as death or new disability, changes from baseline in functional outcomes and health-related quality of life, work status, and persistent symptoms of COVID-19 at 6 months from COVID-19 critical illness.

## Methods

### Study design

The COVID-Recovery study was a multicenter, registry-embedded, prospective cohort study conducted at 30 ICUs in Australia (Additional file 1: Table S1). The study was embedded in the Short Period Incidence Study of Severe Acute Respiratory Infection (SPRINT-SARI) Australia [15].

We had ethical approval at all participating sites, including a waiver of consent for hospital data and an opt-out consent for telephone follow-up at 6 months.

### Setting

ICUs admitting patients with COVID-19 and enrolled in SPRINT-SARI Australia were invited to participate. SPRINT-SARI Australia captured > 95% of all ICU COVID-19 admissions in Australia, and data were also utilized by the Australian Government to produce official fortnightly reports regarding ICU COVID-19 patients [16] (Additional file 1: Fig. S1).

### Patients

Patients were eligible if they were adults (≥ 18 years) with a positive laboratory PCR for SARS-CoV-2 admitted to an Australian ICU for > 24 h. Patients were excluded if they: (1) declined to participate; (2) were unable to communicate via a translation service or in English; (3) were living overseas; or (4) were still in hospital at 6 months.

The overall population of the COVID-Recovery study is defined as the *‘Hospital Cohort’*, comprising all patients who met the criteria above. The main population of interest for this study is patients who died within 6 months of ICU admission or those who were contacted by phone at 6 months, together defined as the *‘Follow-Up Cohort’*. *‘Responders’* was defined as surviving patients with available functional outcomes at 6 months.

### Data collection

Demographic, intervention and hospital outcome data were obtained retrospectively from consecutive patients enrolled in SPRINT-SARI Australia for all eligible patients. We contacted patients prospectively who survived the hospital admission by mail and invited them to participate in telephone interviews at 6 months after ICU admission. Assessments were performed by telephone with trained outcome assessors. We confirmed consent at the start of the interview with patients. We assessed baseline health and disability in the month before ICU admission retrospectively at six months.

### Data definitions

We determined frailty using the Clinical Frailty Scale (CFS) [17, 18] at the time of ICU admission, based on the patient's level of physical function in the month preceding admission [19]. The highest level of mobility during ICU stay was determined using the ICU mobility scale [20]. ICU-acquired weakness was determined at ICU discharge using the Medical Research Council manual muscle test sum-score (MRC MMT-SS) and defined as MRC MMT-SS < 48 [21].

The functional outcome measures recorded at 6 months are described in Additional file 1: Table S2. The primary outcome was death or new disability in survivors at 6 months. Most measurement tools were selected as they are part of a recommended core outcome set for survivors of acute respiratory failure [22]. The World Health Organisation Disability Assessment Schedule 2.0-12L (WHODAS) is reported as a percentage score, with higher percentages representing greater disability [23]. New disability was an increase in WHODAS at 6 months from baseline of 10% or more, and a poor outcome was the combination of new disability or death at 6 months [24]. All components of EQ-5D-5L^TM^, Hospital Anxiety and Depression Scale (HADS), Impact of Events Scale-6 (IES-6), Montreal Cognitive Assessment (MoCA-BLIND), Instrumental Activities of Daily Living (IADL) and work status were reported.

### Statistical analysis

Continuous variables are reported as medians and interquartile ranges (IQR) and categorical variables as number and percentage. Comparison between groups was done using Wilcoxon rank-sum or Kruskal–Wallis tests for continuous variables and Fisher exact test for categorical variables.

Comparison of baseline and 6-month categorical outcomes was done using mixed-effect generalized linear models with binomial distribution and identity link and reported as risk difference (RD). For continuous outcomes, mixed-effect quantile models considering a *Τ* = 0.50 and an interior point algorithm were used and reported as median difference (MD). For the five WHODAS disability categories, a mixed-effect cumulative logistic models were used and reported as common odds ratio (COR). In all models, time was treated as fixed effect, while patients were treated as random effects to account for repeated measurements. All results are reported with 95% confidence intervals (CI). Symptoms at 6 months were compared between patients who developed new disability and those without new disability.

A mixed-effects multivariable logistic regression model was used to identify factors independently associated with death or new disability at 6 months, with results reported as odds ratios (OR) and participating units included as random effects. A list of candidate predictors was determined a priori and included only variables with a known or potential association with the outcome [23, 25–27]. The multivariable model was constructed using a least absolute shrinkage and selection operator approach and confirmed using a backwards elimination technique before undergoing a final assessment for clinical and biological plausibility. Multicollinearity in the final model was assessed using variance-inflation factors. The linearity assumption of continuous variables was assessed through the Box-Tidwell transformation considering the full model, testing the log-odds and the predictor variable. A two-sided *p* value < 0.05 was considered. Long-term outcomes were reported with specific denominators, and patients with missing data were excluded. Missing data in baseline covariates were present in less than 3% of the cases. For the multivariable models, missing values in continuous variables were imputed by the median, while missing values in categorical variables were coded as a new category of ‘unknown’. All analyses were performed using R software, version 4.0.2 (R Core Team) [28].

## Results

### Patients

**Fig. 1:**
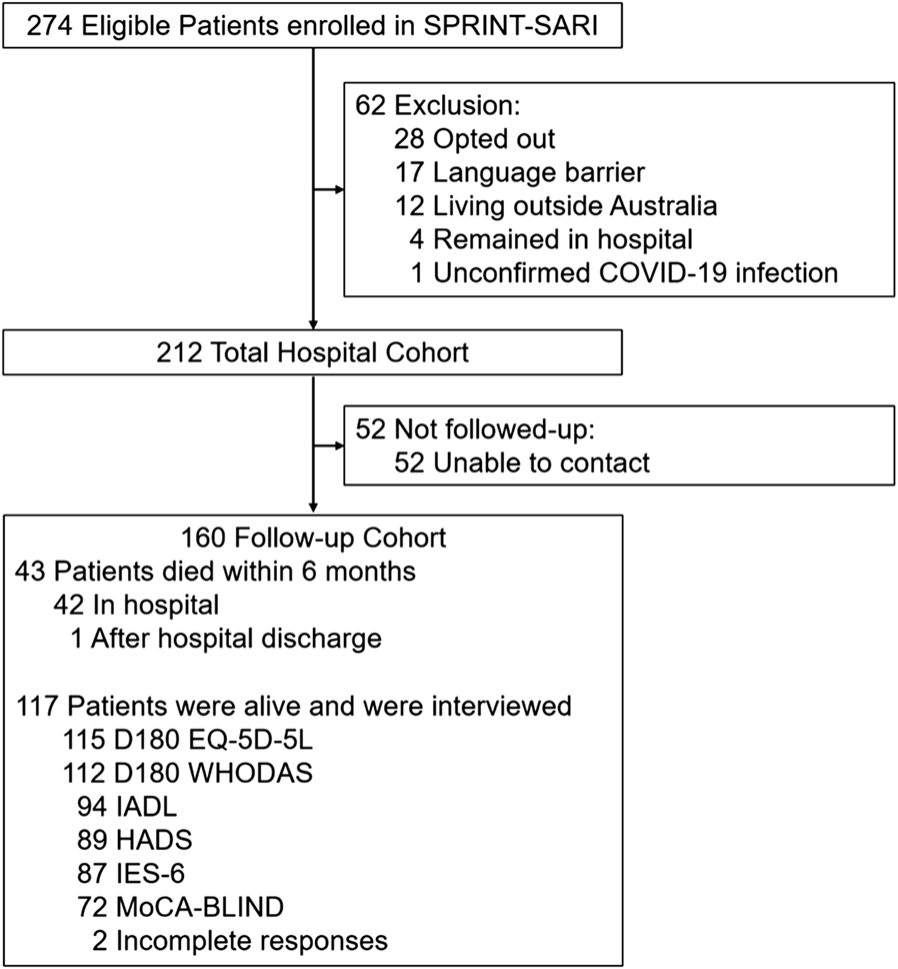
Study flowchart. SPRINT-SARI = WHODAS = The World Health Organization Disability Assessment Schedule 2.0 12 level; HADS = Hospital Anxiety and Depression Scale; IES-6 = Impact of Events Scale-6 questions; MoCA-Blind = Montreal Cognitive Assessment Score-Blind; IADL = Instrumental activities of Daily Living

Between March 06, 2020, and October 04, 2020, 274 critically ill patients with COVID-19 were enrolled in COVID-Recovery from 30 sites in six states of Australia (Fig. 1). Sixty-two patients were excluded; thus, 212 patients were included in the study. Between enrolment and 6-month follow-up, 52 (24.5%) patients were unable or not willing to be contacted at 6 months, leaving 160 (75.5%) patients who were included (Fig. 1). The final 6-month follow-up was conducted on April 21, 2021.

Baseline characteristics of the hospital cohort at hospital admission are shown in Table 1. Overall, median age was 61 (51–70) years, 124 (58.5%) patients were male, and the median clinical frailty score was 3 (2–3). The most prevalent coexisting disorder was diabetes (32.5%) followed by obesity (30.0%). Most of the patients presented at ICU admission with fever (77.9%), cough (70.1%) and shortness of breath (66.2%). At 6 months, 43 of the 160 (26.9%) patients died (42 during the hospital stay and 1 after discharge) (Additional file 1: Fig. S2). Baseline characteristics of the patients not assessed at 6 months are shown in Additional file 1: Table S3.

**Table 1:** Baseline characteristics and clinical outcomes of the included patients

	Hospital cohort (*n* = 212)	Follow-up cohort^a^ (*n* = 160)
*Age, years*	61 (51–70)	62 (55–71)
< 60	99 (46.7)	69 (43.1)
60–69	54 (25.5)	43 (26.9)
70–79	48 (22.6)	39 (24.4)
> 80	11 (5.2)	9 (5.6)
Male gender—no. (%)	124 (58.5)	97 (60.6)
APACHE II	14 (10–19)	15 (11–19)
Days between symptoms—hospital admission	6.1 (3.6–9.2)	6.4 (3.4–9.2)
Days between symptoms—ICU admission	8.1 (5.3–11.0)	8.1 (5.4–11.0)
Body mass index, kg/m^2^	29.4 (25.0–35.7)	30.0 (25.5–35.8)
Clinical frailty score	3 (2–3)	3 (2–3)
Years of education	14 (11–16)	14 (11–16)
Healthcare worker—no. (%)	24/205 (11.7)	15/153 (9.8)
*Coexisting disorders*—*no. (%)*		
Diabetes	67/206 (32.5)	56/154 (36.4)
Obesity	60/200 (30.0)	48/151 (31.8)
Use of ACEi or ARB	38/198 (19.2)	31/148 (20.9)
Chronic cardiac failure	35/202 (17.3)	29/152 (19.1)
Smoker	25/197 (12.7)	18/148 (12.2)
Chronic pulmonary disease**	16/201 (8.0)	14/153 (9.2)
Asthma	28/202 (13.9)	18/153 (11.8)
Immunosuppression	24/202 (11.9)	20/152 (13.2)
Chronic kidney disease	14/203 (6.9)	12/153 (7.8)
Chronic hematological disease	9/202 (4.5)	8/152 (5.3)
Cancer	12/203 (5.9)	11/153 (7.2)
*Symptoms*—*no. (%)*		
Fever	159/204 (77.9)	119/152 (78.3)
Cough	143/204 (70.1)	108/152 (71.1)
Shortness of breath	135/204 (66.2)	101/152 (66.4)
Fatigue	106/204 (52.0)	81/152 (53.3)
Myalgia	73/204 (35.8)	63/152 (41.4)
Diarrhea	59/204 (28.9)	42/152 (27.6)
Sore throat	46/204 (22.5)	35/152 (23.0)
Anosmia	22/204 (10.8)	16/152 (10.5)
Runny nose	22/204 (10.8)	17/152 (11.2)
*Signs at baseline*		
Heart rate, bpm	100 (85–110)	99 (85–110)
Respiratory rate, breaths/min	30 (24–38)	30 (24–38)
Mean arterial pressure, mmHg	81 (70–96)	81 (70–97)
Temperature, °C	38.3 (37.4–38.9)	38.2 (37.4–38.8)
SpO_2_, %	92 (88–95)	92 (88–95)
*Clinical outcomes*		
Hospital readmission—no. (%)	–	18/108 (16.7)
Duration of ventilation, days	12 (5–19)	13 (5–19)
ICU length of stay, days	6.9 (3.2–17.1)	8.3 (3.6–19.0)
Hospital length of stay, days	16.1 (9.6–26.7)	16.9 (10.1–30.4)
ICU mortality—no. (%)	39 (18.4)	39 (24.4)
Hospital mortality—no. (%)	42/211 (19.9)	42 (26.2)
Death or new disability at 6 months—no. (%)	–	85/151 (56.3)

Data are median (quartile 25%–quartile 75%) or no (%). Percentages may not total 100 because of rounding

*APACHE* Acute Physiology and Chronic Health Evaluation, *ACEi* angiotensin-converting enzyme inhibitor, *ARB* angiotensin II receptor blocker, *ICU* intensive care unit

^a^The follow-up cohort comprises patients who died within 6 months or who were contacted successfully at 6 months. However, data may be missing for some outcomes depending on the willingness of the patient to answer all questions

^*^On a scale from 1 to 10 (1 would be the lowest level of financial distress)

^**^Not considering asthma

During the hospital stay, among 212 patients, 188 (91.7%) of the patients were treated with antibiotics and 147 (71.7%) with steroids (Additional file 1: Table S4). Overall, 120 (57.1%) patients received mechanical ventilation, 112 (55.2%) inotropes or vasopressors, 129 (61.6%) were treated with high-flow nasal cannula, and 29 (14.3%) with non-invasive ventilation. The median highest ICU mobility scale achieved while in the ICU was 5 (3–8), and the MRC MMT-SS at ICU discharge was 48 (34–60), with ICU-acquired weakness present in 58 of 135 (42.3%) patients. Treatment characteristics of the *‘Follow-Up Cohort’* followed the same pattern, and characteristics of the patients not assessed at 6 months are shown in Additional file 1: Table S5.

### Baseline function in the follow-up cohort

**Fig. 2:**
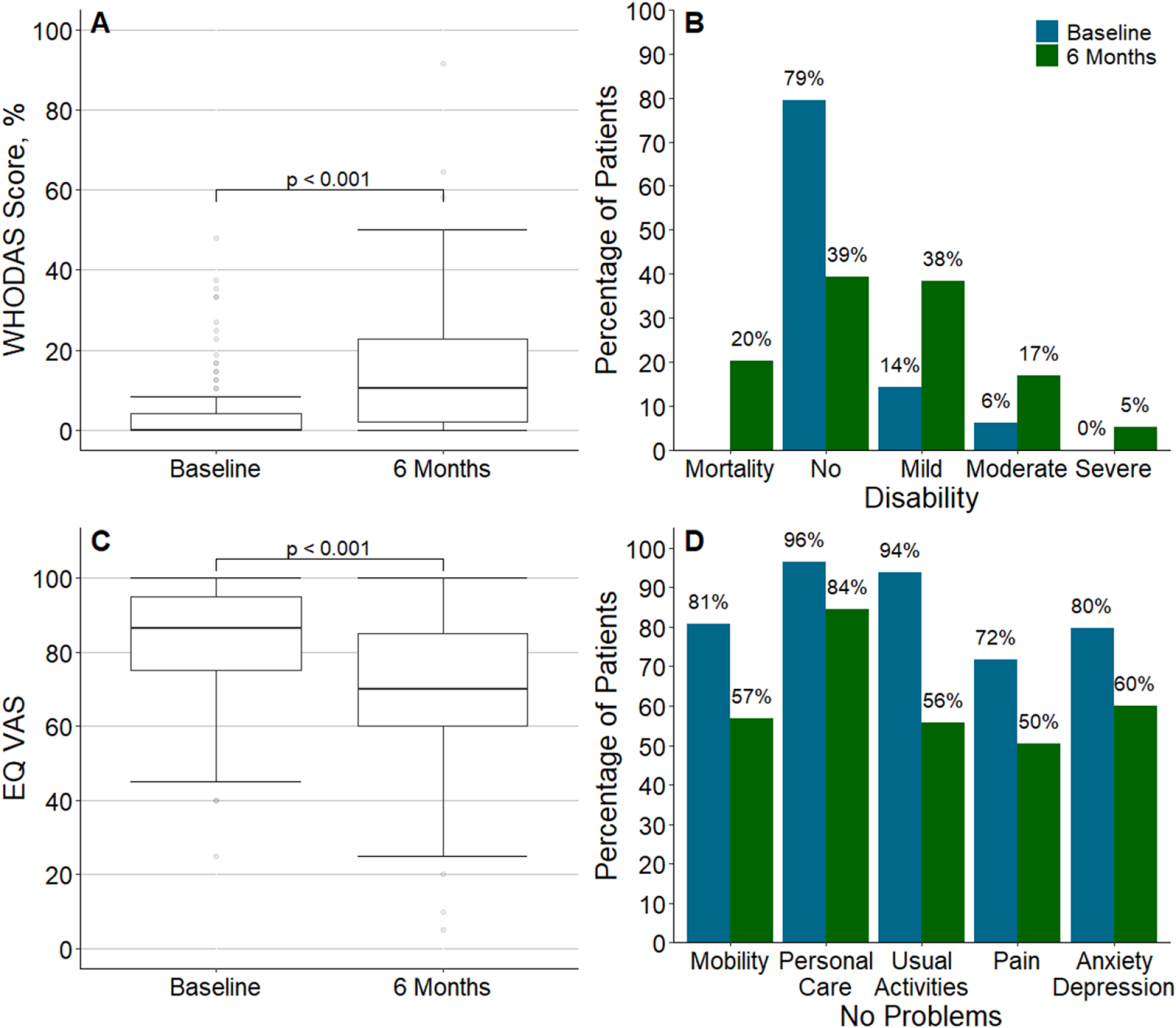
WHODAS Score and EQ-5D-5L Scale. **a** Comparison of WHODAS score at baseline and 6 months (*p* value from a mixed-effect quantile models considering a Τ = 0.50, an interior point algorithm, and including patients as random effect). **b** Proportion of patients who have died or who have developed no (WHODAS < 5%), mild (5% ≤ WHODAS < 25%), moderate (25% ≤ WHODAS < 50%) or severe (50% ≤ WHODAS < 96%) disability. *P* values for comparisons are shown in Table 2. **c** Comparison of health status using the EQ-VAS at baseline and 6 months (*p* value from a mixed-effect quantile models considering a *Τ* = 0.50, an interior point algorithm, and including patients as random effect). **d** the domains of the EQ5D-5L, including the proportion of patients who reported no problems with mobility, personal care, usual activities, pain/discomfort or anxiety/depression. *P* values for comparisons are shown in Table 2

Among patients alive at 6 months and with baseline function available, the median WHODAS score was 0% (0–4%), with 7/112 (6.2%) patients presenting with moderate disability and no patients with severe disability. The median EQ5D visual analogue scale (EQ-VAS) was median 86 (75–95), and the EQ-5D-5L utility score was 1.0 (0.8–1.0). Overall, 92/114 (80.7%) patients did not report any problems with mobility, 109/113 (96.5%) did not report any problems with personal care, 106/113 (93.8%) did not report any problem with usual activities, 81/113 (71.7%) did not report any problem with pain or discomfort, and 90/113 (79.6%) did not report any problem with anxiety or depression (Table 2 and Fig. 2).

**Table 2:** Functional outcomes at baseline and 6 months

	Baseline (*n* = 115)	6 months (*n* = 115)	Absolute difference (95% CI)	*p* value
Treatment for anxiety or depression—no. (%)	13/108 (12.0)	16/109 (14.7)	RD, 2.21 (− 3.75 to 8.20)	0.467
Unemployed due to health reasons—no. (%)	–	13/114 (11.4)	–	–
*EQ-5D-5L*				
Utility score	1.0 (0.8–1.0)	0.8 (0.7–0.9)	MD, − 0.19 (− 0.28 to − 0.10)	< 0.001
No problems with mobility	92/114 (80.7)	66/115 (57.3)	RD, − 23.92 (− 32.96 to − 14.87)	< 0.001
New problems with mobility*	–	38/112 (33.9)	–	–
No problems with personal care	109/113 (96.5)	97/115 (84.3)	RD, − 12.00 (− 18.52 to − 5.50)	< 0.001
New problems with personal care*	–	15/111 (13.5)	–	–
No problems with usual activities	106/113 (93.8)	64/115 (55.7)	RD, − 38.11 (− 47.79 to − 28.45)	< 0.001
New problems with usual activities*	–	48/111 (43.2)	–	–
No problems with pain/discomfort	81/113 (71.7)	58/115 (50.4)	RD, − 21.06 (− 31.08 to − 11.06)	< 0.001
New problems with pain/discomfort*	–	38/111 (34.2)	–	–
No problems with anxiety/depression	90/113 (79.6)	69/115 (60.0)	RD, − 19.26 (− 27.38 to − 11.16)	< 0.001
New problems with anxiety/depression*	–	29/111 (26.1)	–	–
EuroQol-visual analogue scale	86.5 (75.0–95.0)	70.0 (60.0–85.0)	MD, − 15.00 (− 21.04 to − 8.95)	< 0.001
*Marital status*			–	–
Never married	–	19/113 (16.8)		
Currently married	–	65/113 (57.5)		
Separated	–	3/113 (2.7)		
Divorced	–	8/113 (7.1)		
Widowed	–	9/113 (8.0)		
Cohabiting	–	9/113 (8.0)		
Financial distress**	1 (1–4)	1 (1–5)	MD, 0.00 (− 1.07 to 1.07)	0.999
*WHODAS score, %*	0.0 (0.0–4.2)	10.4 (2.1–22.9)	MD, 10.40 (7.06–13.77)	< 0.001
Disability—no. (%)	7/112 (6.2)	25/112 (22.3)	RD, 16.01 (7.90–24.12)	< 0.001
New disability—no. (%)	–	42/108 (38.9)	–	–
Category—no. (%)				
No disability	89/112 (79.5)	44/112 (39.3)	COR, 11.22 (4.94–25.52)	< 0.001
Mild disability	16/112 (14.3)	43/112 (38.4)		
Moderate disability	7/112 (6.2)	19/112 (17.0)		
Severe disability	0/112 (0.0)	6/112 (5.4)		
Complete disability	0/112 (0.0)	0/112 (0.0)		
*IADL*	–	8.0 (7.0–8.0)	–	–
Fully independent (IADL = 8)	–	66/94 (70.2)	–	–
*HADS anxiety*	–	3.0 (1.0–7.0)	–	–
Anxiety (HADS ≥ 8)	–	18/89 (20.2)	–	–
*HADS depression*	–	4.0 (1.0–7.0)	–	–
Depression (HADS ≥ 8)	–	18/89 (20.2)	–	–
*IES-6 total*	–	4.0 (1.0–9.0)	–	–
Mean score	–	0.7 (0.2–1.5)	–	–
Post-traumatic stress disorder (IES-6 mean > 1.75)	–	16/87 (18.4)	–	–
*MoCA-BLIND*	–	19.0 (17.0–20.0)	–	–
Cognitive dysfunction (MoCA-BLIND < 18)	–	24/72 (33.3)	–	–

Data are median (quartile 25%–quartile 75%) or no (%). Percentages may not total 100 because of rounding

*CI* confidence interval, *COR* common odds ratio where > 1.00 represents a higher chance of being in a worse category at 6 months compared to baseline, *MD* median difference, *RD* risk difference

^*^New problems defined when the score of the specific component of EQ-5D-5L at 6 months was higher than at baseline

^**^On a scale from 1 to 10 (1 would be the lowest level of financial distress)

### Functional outcomes at 6 months in the follow-up cohort

**Fig. 3:**
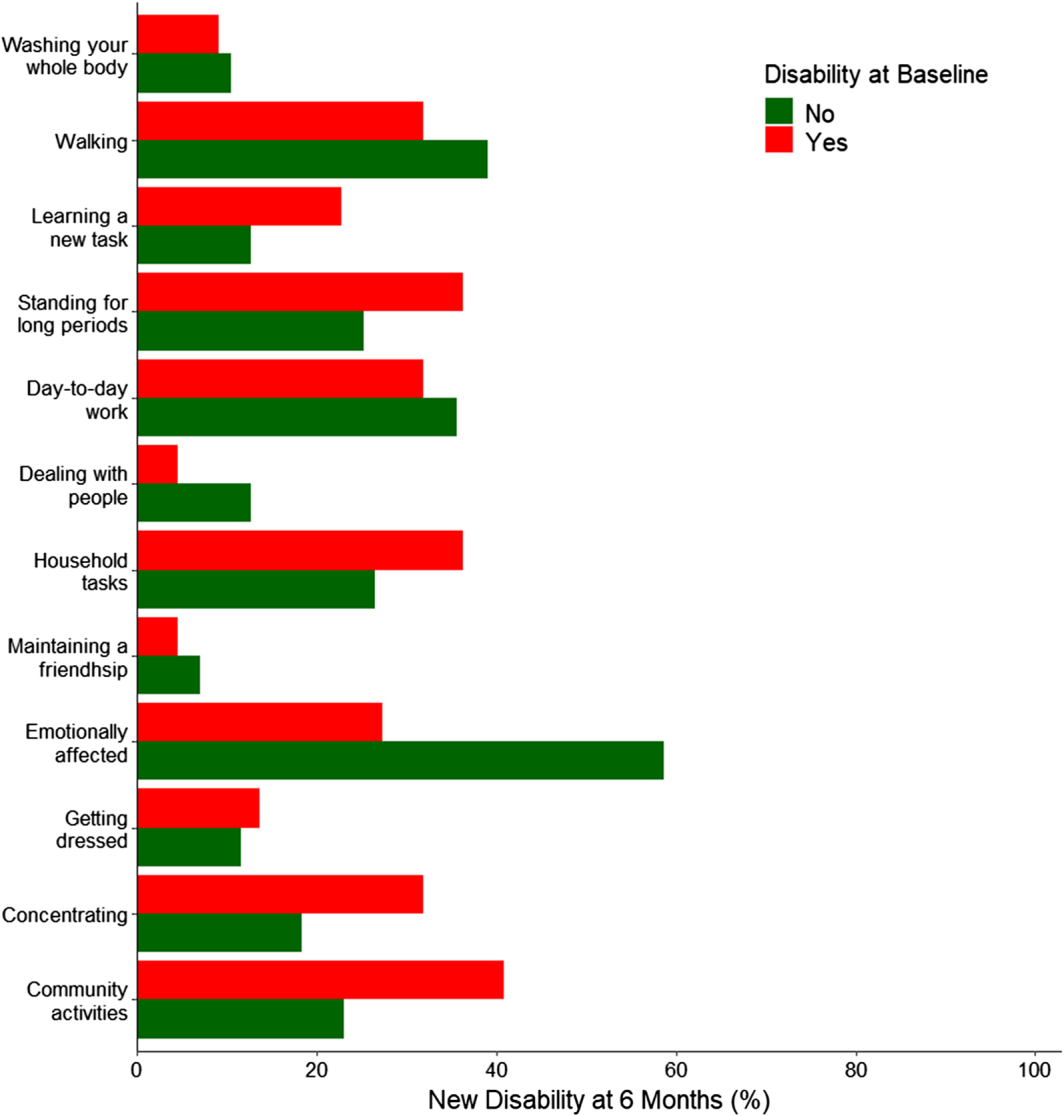
New disability at 6 months according to baseline disability. Proportion of patients with new disability compared to baseline disability. No single item of the WHODAS 2.0 12L accounted for new disability. There was a wide range of new disabilities across all items

For the primary outcome, there was a total of 43/160 (26.9%) patients who died and 42/108 (38.9%) with new disability. The WHODAS percentage score was significantly higher at 6 months compared to baseline (MD, 10.40% [95% CI 7.06–13.77]; *p* < 0.001). The severity of disability (COR, 11.22 [95% CI 4.94–25.52]; *p* < 0.001) increased. 13/114 (11.4%) survivors were unable to return to work due to poor health (Table 2 and Fig. 2). Components of WHODAS score at 6 months are shown in Additional file 1: Table S6. New disability according to baseline disability is shown in Fig. 3. No single item of the WHODAS accounted for new disability, with a wide range of new disabilities across all items. Survivors without baseline disability were more likely to have new disability in walking, day-to-day work and being emotionally affected.

There was a decrease in the EQ-VAS (MD, − 15.00 [95% CI − 21.04 to − 8.95]; *p* < 0.001), in the EQ-5D-5L utility score (MD, − 0.19 [− 0.28 to − 0.10]; *p* < 0.001), and in the proportion of patients reporting no problems in all domains (Table 2 and Fig. 2). New problems were reported across all domains, but mostly with usual activities, pain and mobility in 48/111 (43.2%), 38/111 (34.2%) and 38/112 (33.9%) of the patients, respectively. While 66/94 (70.2%) patients were fully independent as measured with the IADL, 18/89 (20.2%) presented with some degree of anxiety and/or depression, 16/87 (18.4%) had a positive screen for PTSD, and 24/72 (33.3%) had some degree of cognitive dysfunction (Table 2). There was no difference in the percentage of patients receiving treatment for anxiety or depression from before the critical illness (RD, 2.21 [95% CI − 3.75 to 8.20]; *p* = 0.467) or in financial distress (MD, 0.00 [95% CI − 1.07 to 1.07]; *p* = 0.999).

### Persistent symptoms at 6 months in the follow-up cohort

**Fig. 4:**
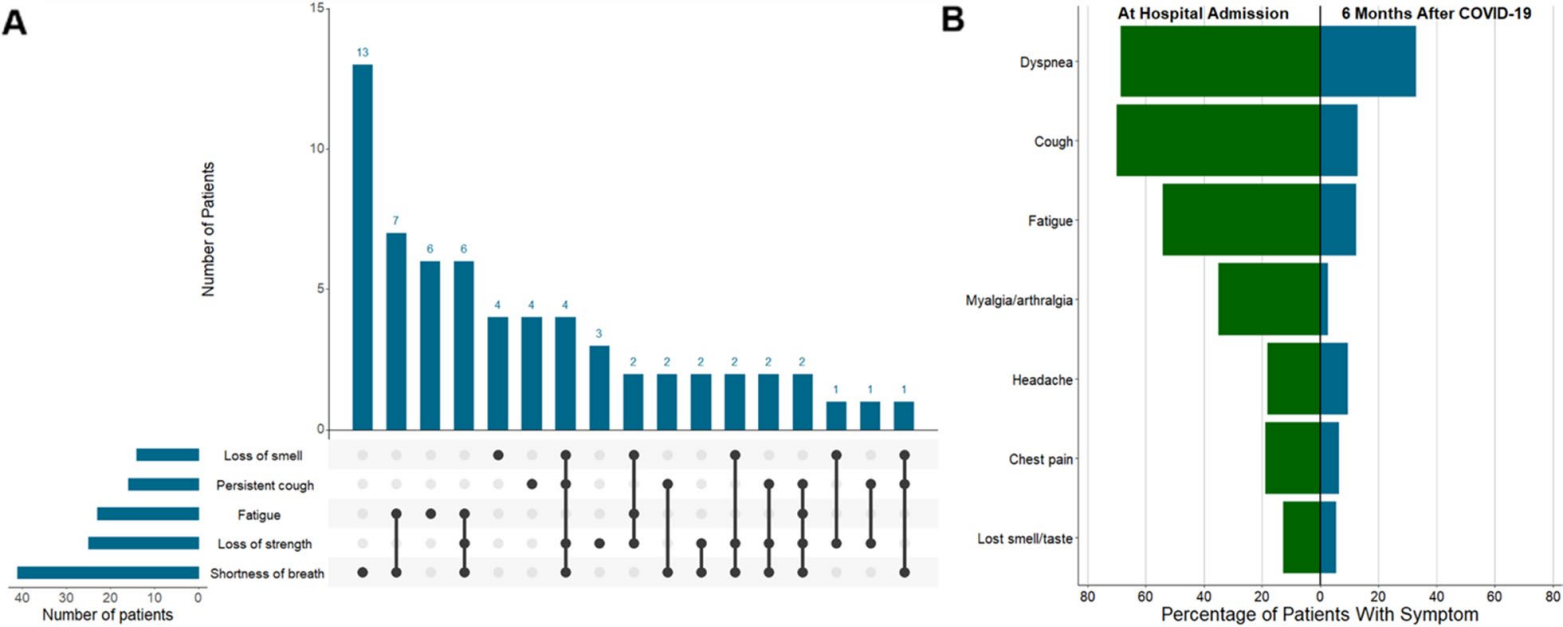
Persistent symptoms at 6 months. **a** Intersection plot showing the most prevalent symptoms and combinations of symptoms at 6 months. **b** Symptoms at acute phase (at hospital admission) and after 6 months of COVID-19. Fatigue combines fatigue and/or loss of strength

Among 115 surviving patients at 6 months, 82 (71.3%) of the patients reported persistent symptoms, with 50 (43.5%) reporting three or more symptoms (Table 3). The most frequent symptom was shortness of breath (34.8%), followed by loss of strength (21.7%) and fatigue (19.1%). Patients who developed new disability at 6 months also had more symptoms at 6 months, including a higher prevalence of shortness of breath (61.9% vs. 21.2%; *p* < 0.001) and loss of strength (45.2% vs. 9.1%; *p* < 0.001) (Table 3). The most frequent combination of symptoms was shortness of breath and fatigue followed by shortness of breath, loss of strength and fatigue (Fig. 4). Comparisons of symptoms at hospital admission and 6 months after COVID-19 are shown in Fig. 4.

**Table 3:** Symptoms at 6 months in the included patients

	Responders^a^ (*n* = 115)	New disability		
		Yes (*n* = 42)	No (*n* = 66)	*p* value
Any symptom—no. (%)	82 (71.3)	34 (81.0)	46 (69.7)	0.261
*Number of symptoms*—*no. (%)*				0.001
1	23 (20.0)	3 (7.1)	20 (30.3)	
2	9 (7.8)	3 (7.1)	6 (9.1)	
≥ 3	50 (43.5)	28 (66.7)	20 (30.3)	
*Symptoms*—*no. (%)*				
Shortness of breath	40 (34.8)	26 (61.9)	14 (21.2)	< 0.001
Loss of strength	25 (21.7)	19 (45.2)	6 (9.1)	< 0.001
Fatigue	22 (19.1)	11 (26.2)	10 (15.2)	0.213
Persistent cough	16 (13.9)	10 (23.8)	6 (9.1)	0.051
Loss of taste	14 (12.2)	9 (21.4)	5 (7.6)	0.044
Loss of smell	14 (12.2)	9 (21.4)	4 (6.1)	0.030
Headache	12 (10.4)	7 (16.7)	3 (4.5)	0.045
Persistent chest pain	8 (7.0)	5 (11.9)	2 (3.0)	0.107
Palpitations	8 (7.0)	4 (9.5)	4 (6.1)	0.709
Myalgia/arthralgia	8 (7.0)	3 (7.1)	5 (7.6)	0.999
Loss of sensation	7 (6.1)	4 (9.5)	3 (4.5)	0.427
Hair loss	6 (5.2)	3 (7.1)	3 (4.5)	0.676
Weight loss	6 (5.2)	3 (7.1)	3 (4.5)	0.676
Anxiety	5 (4.3)	3 (7.1)	2 (3.0)	0.375
Other	28 (24.3)	15 (35.7)	12 (18.2)	0.067

Data are no (%). Percentages may not total 100 because of rounding

^a^The follow-up cohort comprises patients who were contacted successfully at 6 months. However, data may be missing for some outcomes depending on the willingness of the patient to answer all questions

In the period after hospital discharge to 6 months, 56/122 (46.7%) survivors did not seek further outpatient multidisciplinary support. There were 40/122 (32.8%) who attended physical therapy, 9/122 (7.5%) who accessed psychology and 4/122 (3.3%) who saw a dietician.

### Predictors of new disability or death

In the unadjusted analyses, higher age, higher APACHE II score, higher clinical frailty score, presence of diabetes, chronic cardiac failure or cancer, degree of respiratory support, use of neuromuscular blocking agents and use of prone positioning were associated with new disability or death (Additional file 1: Table S7). After variable selection, in the multivariable model only higher APACHE II (OR, 1.08 [95% CI 1.01–1.17]; *p* = 0.032) and greater clinical frailty at baseline (OR, 1.49 [95% CI 1.05–2.11]; *p* = 0.025) were independently associated with new disability or death.

## Discussion

In this nationally representative cohort of critically ill patients with COVID-19, at 6 months, 26.9% patients died and 38.9% survivors reported new disability. In survivors, disability was widespread across all areas of functioning. There was a significant decrease in health-related quality of life, with over one third of the cohort reporting new problems in mobility, usual activities and pain. In addition, one third of survivors had cognitive impairment and one fifth of the cohort reported anxiety, depression and/or PTSD. More than one in ten survivors were unemployed due to poor health. Higher severity of illness and the clinical frailty score were independent predictors of death or new disability. The majority of our cohort (70%) had ongoing symptoms of COVID-19 at 6 months, most commonly shortness of breath, weakness or fatigue.

While the long-term effects of critical illness are well-recognized [23, 29, 30], the scope and scale of “Long COVID” may be greater than previously described in survivors of COVID-19. During the COVID-19 pandemic, the Australian government enacted several healthcare policies that may have influenced the characteristics and outcomes of this group of patients compared with patients from other countries. Australia has a liberal testing policy, and until November 2020, Australia has conducted 9,670,186 COVID-19 tests, representing 377,527 tests per 1,000,000 population and with a positive rate of 0.3% [16]. As a comparison, the entire USA has a rate of 533,967 tests per 1,000,000 population, with a positive rate of 6.9% [31]. The healthcare system in Australia has not been overwhelmed due to COVID-19, and the outcomes of our survivors represent a cohort provided with care from a critical care system operating within capacity [15]. Despite this, the present data are similar to other data of COVID-19 patients in intensive care in terms of age, comorbid conditions and ARDS severity [15, 32], and suggest that COVID-19 survivorship was associated with substantial new disability and reduced health-related quality of life. Increased disability, both in the number of patients and in the severity of functional limitations, are associated with increased caregiver burden, unemployment, psychological problems, mortality and healthcare costs [23, 29, 30]. Patients should be screened at hospital discharge for new functional impairments. Outpatient follow-up should be recommended early, within the first few weeks of discharge. It should include medication optimization and screening for physical, psychological or cognitive problems, with referral for additional services such as physical therapy or psychology as required [11]. In the present study, new disability was present in all areas of function, particularly emotionally (such as anxiety, depression, PTSD) and walking.

We used the WHODAS, a validated outcome measure for disability, grounded in the framework of the International Classification of Functioning (ICF) which has previously been used to described the critically ill population with a defined minimum clinically important difference [23, 24, 33, 34]. The baseline disability of this relatively young cohort was very low prior to COVID-19, and there was a clinically significant increase in the level of disability at 6 months.

Recently, the COMEBAC Investigators have reported the 4-month outcomes of 478 hospitalized patients with COVID-19 in a single center in France [27]. Of this cohort, 142 had been critically ill and approximately 50% had been mechanically ventilated, similar to the present study. New onset dyspnea was one of the most common symptoms, and lung CT scan in survivors showed persistent abnormalities in 75% who had received invasive ventilation. Similarly, in a recent single-center cohort study in China, nearly one third of the 122 critically ill patients with COVID-19 had a mean 6-min walking distance less than the lower limit of the normal range at 6 months after hospitalization [35]. In addition, 56% had diffusion impairment on pulmonary function tests. The results of both these studies are aligned with the high prevalence of shortness of breath in survivors of our cohort. Pulmonary rehabilitation in patients with ongoing shortness of breath may improve outcomes and reduce symptoms [36]. Further, pulmonary rehabilitation may be delivered by telehealth [37–39], improving the access to care during a pandemic.

The strengths of this study include its prospective, multicenter design with collection of detailed clinical and physiologic parameters. We included baseline measures of frailty, health-related quality of life, disability and comorbidities to distinguish new disability and new problems. The outcome measures include validated, reliable measures of function, most of which are in a core outcome set for survivors of acute respiratory failure [22].

We acknowledge limitations to our study. A proportion of eligible patients were not available for follow-up assessment, mainly due to loss to follow-up. This was higher than similar studies of disability at 6 months from our group, and we speculate that it may be due to stigma or psychological distress associated with a positive diagnosis of COVID-19 which should be investigated further in future studies. We contacted primary practitioners and reviewed national online resources for death notices to ensure they were not deceased. The responders had similar baseline characteristics and interventions to the non-responders, and it is likely a good representation of the overall cohort. Baseline disability and health-related quality of life were measured retrospectively in survivors, which may introduce recall bias. There was no control group, and the outcomes of survivors of COVID-19 critical illness from this study may be similar to disability reported after critical illness from other cohorts [23, 25]. We did not conduct in-person assessments or radiological tests as part of the follow-up which would improve the understanding of sequelae of COVID-19.

## Conclusion

In this national cohort of patients with COVID-19 critical illness, death and new disability was common. Over a third of survivors had new disability, which was widespread across all areas of functioning. In survivors who reported new disability, most reported three or more ongoing symptoms at 6 months. These observations suggest that the burden of new disability after COVID-19 represents an urgent public health problem.

## Supplementary Information

**Additional file 1:** Electronic Supplementary Appendix to The COVID-Recovery Study.

## Data Availability

Partial data set sharing according to individual requests for data access. Requests will be considered by the study's Management Committee. Requests for data sharing to be made to anzicrc@monash.edu and the corresponding author, carol.hodgson@monash.edu.

## Code availability

Statistical code is available on request to anzicrc@monash.edu and the corresponding author, carol.hodgson@monash.edu.

## References

[1] Johns Hopkins University of Medicine Coronavirus Resource Center. COVID-19 Dashboard by the Center for Systems Science and Engineering (CSSE) at Johns Hopkins University of Medicine Coronavirus Resource Center. https://coronavirus.jhu.edu/map.html. Accessed 26 May 2021.

[2] Zeng H, Ma Y, Zhou Z, Liu W, Huang P, Jiang M (2021). Spectrum and clinical characteristics of symptomatic and asymptomatic coronavirus disease 2019 (COVID-19) with and without pneumonia. Front Med (Lausanne)..

[3] Del Rio C, Collins LF, Malani P (2020). Long-term health consequences of COVID-19. JAMA.

[4] Cortinovis M, Perico N, Remuzzi G (2021). Long-term follow-up of recovered patients with COVID-19. Lancet.

[5] Fraser E (2020). Long term respiratory complications of covid-19. BMJ.

[6] Sivan M, Rayner C, Delaney B (2021). Fresh evidence of the scale and scope of long covid. BMJ.

[7] Aucott JN, Rebman AW (2021). Long-haul COVID: heed the lessons from other infection-triggered illnesses. Lancet.

[8] Shah W, Heightman M, O'Brien S (2021). UK guidelines for managing long-term effects of COVID-19. Lancet.

[9] Lancet T (2020). Facing up to long COVID. Lancet.

[10] Nabavi N (2020). Long covid: How to define it and how to manage it. BMJ.

[11] Prescott HC, Girard TD (2020). Recovery from severe COVID-19: Leveraging the lessons of survival from sepsis. JAMA.

[12] Needham DM, Davidson J, Cohen H, Hopkins RO, Weinert C, Wunsch H (2012). Improving long-term outcomes after discharge from ICU: report from a stakeholders' conference. Crit Care Med.

[13] Norton A, Olliaro P, Sigfrid L, Carson G, Paparella G, Hastie C (2021). Long COVID: tackling a multifaceted condition requires a multidisciplinary approach. Lancet Infect Dis.

[14] Yelin D, Wirtheim E, Vetter P, Kalil AC, Bruchfeld J, Runold M (2020). Long-term consequences of COVID-19: research needs. Lancet Infect Dis.

[15] Burrell AJ, Pellegrini B, Salimi F, Begum H, Broadley T, Campbell LT (2021). Outcomes for patients with COVID-19 admitted to Australian intensive care units during the first four months of the pandemic. Med J Aust.

[16] Australian Government Department of Health and Ageing. Coronavirus disease (COVID-19) epidemiology reports, Australia, 2020. https://www1.health.gov.au/internet/main/publishing.nsf/Content/novel_coronavirus_2019_ncov_weekly_epidemiology_reports_australia_2020.htm. Accessed 30 Aug 2021.

[17] Bagshaw SM, Stelfox HT, McDermid RC, Rolfson DB, Tsuyuki RT, Baig N (2014). Association between frailty and short- and long-term outcomes among critically ill patients: a multicentre prospective cohort study. CMAJ.

[18] Rockwood K, Song X, MacKnight C, Bergman H, Hogan DB, McDowell I (2005). A global clinical measure of fitness and frailty in elderly people. CMAJ.

[19] Utino Taniguchi L, Ibrahim Q, Azevedo LCP, Stelfox HT, Bagshaw SM (2020). Comparison of two frailty identification tools for critically ill patients: a post-hoc analysis of a multicenter prospective cohort study. J Crit Care.

[20] Hodgson CL (2014). Feasibility and inter-rater reliability of the ICU mobility scale. Heart Lung..

[21] Hodgson CL, Tipping CJ (2017). Physiotherapy management of intensive care unit-acquired weakness. J Physiotherapy..

[22] Needham DM, Sepulveda KA, Dinglas VD, Chessare CM, Aronson Friedman L, Bingham Iii CO (2017). Core outcome measures for clinical research in acute respiratory failure survivors: an international modified Delphi consensus study. Am J Respir Crit Care Med.

[23] Hodgson CL, Udy AA, Bailey M, Barrett J, Bellomo R, Bucknall T (2017). The impact of disability in survivors of critical illness. Intensive Care Med.

[24] Higgins AM, Serpa Neto A, Bailey M, Barrett J, Bellomo R, Cooper DJ (2021). The psychometric properties and the minimal clinically important difference for disability assessment using the WHODAS 2.0 in critically ill patients. Crit Care Res.

[25] Higgins AM, Neto AS, Bailey M, Barrett J, Bellomo R, Cooper DJ (2021). Predictors of death and new disability after critical illness: a multicentre prospective cohort study. Intensive Care Med.

[26] Miskowiak KW, Johnsen S, Sattler SM, Nielsen S, Kunalan K, Rungby J (2021). Cognitive impairments four months after COVID-19 hospital discharge: pattern, severity and association with illness variables. Eur Neuropsychopharmacol.

[27] Morin L, Savale L, Pham T, Colle R, Figueiredo S, Writing Committee for the COMEBAC Study Group (2021). Four-month clinical status of a cohort of patients after hospitalization for COVID-19. JAMA.

[28] R Core Team. R: a language and environment for statistical computing. R Foundation for Statistical Computing, Vienna, Austria; 2019. https://www.R-project.org/. Accessed 26 May 2021.

[29] Herridge MS, Cheung AM, Tansey CM, Matte-Martyn A, Diaz-Granados N, Al-Saidi F (2003). One-year outcomes in survivors of the acute respiratory distress syndrome. N Engl J Med.

[30] Herridge MS, Tansey CM, Matte A, Tomlinson G, Diaz-Granados N, Cooper A (2011). Functional disability 5 years after acute respiratory distress syndrome. N Engl J Med.

[31] Worldometer. COVID-19 coronavirus pandemic. Worldometer; 2021. https://www.worldometers.info/coronavirus/. Accessed 2021 August 30.

[32] Grasselli G, Zangrillo A, Zanella A, Antonelli M, Cabrini L, Castelli A (2020). Baseline characteristics and outcomes of 1591 patients infected with SARS-CoV-2 admitted to ICUs of the Lombardy Region. Italy JAMA.

[33] Ustun TB, Chatterji S, Kostanjsek N, Rehm J, Kennedy C, Epping-Jordan J (2010). Developing the World Health Organization Disability Assessment Schedule 2.0. Bull World Health Organ.

[34] Higgins AM, Serpa Neto A, Bailey M, Barrett J, Bellomo R, Cooper DJ (2021). Predictors of death and new disability after critical illness: a multicentre prospective cohort study. Intensive Care Med.

[35] Huang C, Huang L, Wang Y, Li X, Ren L, Gu X (2021). 6-month consequences of COVID-19 in patients discharged from hospital: a cohort study. Lancet.

[36] Alison JA, McKeough ZJ, Johnston K, McNamara RJ, Spencer LM, Jenkins SC (2017). Australian and New Zealand pulmonary rehabilitation guidelines. Respirology.

[37] Cox NS, Dal Corso S, Hansen H, McDonald CF, Hill CJ, Zanaboni P (2021). Telerehabilitation for chronic respiratory disease. Cochrane Database Syst Rev.

[38] Holland AE, Cox NS (2017). Telerehabilitation for COPD: could pulmonary rehabilitation deliver on its promise?. Respirology.

[39] Wootton SL, King M, Alison JA, Mahadev S, Chan ASL (2020). COVID-19 rehabilitation delivered via a telehealth pulmonary rehabilitation model: a case series. Respirol Case Rep..

