# Supplementary material for: The impact of COVID-19 critical illness on new disability, functional outcomes and return to work at 6 months: a prospective cohort study
Source: Crit Care. 2021 Nov 8;25:382. doi: 10.1186/s13054-021-03794-0 (PMC8575157; doi:10.1186/s13054-021-03794-0)
Supplement: Supplementary file 1 — Additional file 1: Electronic Supplementary Appendix to The COVID-Recovery Study. [file 13054_2021_3794_MOESM1_ESM.pdf]

ONLINE SUPPLEMENT

The Impact of COVID-19 Critical Illness on Disability, Functional Outcomes and Return to Work at 6 Months

The COVID-Recovery Study Investigators and the ANZICS Clinical Trials Group

The COVID-Recovery Study Investigators and the ANZICS Clinical Trials Group

Carol L. Hodgson, PhD[1,2,3]; Alisa M. Higgins, PhD[1]; Michael J. Bailey, PhD[1]; Anne M. Mather, BBiomed(Hons)[1]; Lisa Beach, MPhysio(Cardio)[3]; Rinaldo Bellomo, PhD[1,11,26]; Bernie Bissett, PhD[6,7]; Ianthe J. Boden, PhD[8,9]; Scott Bradley, PhD[3]; Aidan Burrell, PhD [1,2]; D. James Cooper, MD[1,2]; Bentley J. Fulcher, BBiomedSC(PharmSc)(Hons)[1]; Kimberley J. Haines, PhD[10,11]; Jack Hopkins[1]; Alice Y.M. Jones, PhD[12]; Stuart Lane, PhD[1,13]; Drew Lawrence, BPhy[3]; Lisa van der Lee, PhD[14]; Jennifer Liacos, BPhty(Hons)[1,3]; Natalie J. Linke, BN[1]; Lonni Marques Gomes, BAccMgt[1]; Marc Nickels, PhD[15]; George Ntoumenopoulos, PhD[16]; Paul S. Myles, DSc[17]; Shane Patman, PhD[18]; Michelle Paton, MPhty[1,19]; Gemma Pound, BSc(Phty)(Hons)[1,20]; Sumeet Rai, MBBS[21,22]; Alana Rix, BPhysio(Hons)[3]; Thomas C. Rollinson, BPhysio(Hons)[23,24]; Janani Sivasuthan, MPH[1]; Claire J. Tipping, PhD[3]; Peter Thomas, PhD[25]; Tony Trapani, BEmergHealth(Pmed)[1]; Andrew A. Udy, PhD[1,2]; Christina Whitehead, MBioethics[1,13]; Shannah Anderson (consumer representative); Ary Serpa Neto, PhD[1,5,26,27]

Affiliations

[1] Australian and New Zealand Intensive Care Research Centre, School of Public Health and Preventive Medicine, Monash University, Melbourne, Victoria, Australia

[2] Department of Intensive Care and Hyperbaric Medicine, The Alfred, Melbourne, Victoria, Australia

[3] Department of Physiotherapy, The Alfred, Melbourne, Victoria, Australia

[4] Department of Physiotherapy (Allied Health), The Royal Melbourne Hospital, Melbourne, Victoria, Australia

[5] Department of Critical Care, School of Medicine, University of Melbourne, Victoria, Australia

[6] Discipline of Physiotherapy, University of Canberra, Canberra, Australia

[7] Physiotherapy Department, Canberra Hospital, Canberra, Australia

[8] Physiotherapy Department Launceston General Hospital, Launceston, Australia

[9] Launceston Clinical School, University of Tasmania, Tasmania, Australia

[9] Faculty of Medicine, Nursing and Health Sciences, Monash University, Melbourne, Victoria, Australia

[10] Physiotherapy Department, Western Health, Melbourne, Victoria, Australia

[11] Department of Critical Care, School of Medicine, University of Melbourne, Melbourne, Victoria, Australia

[12] School of Health and Rehabilitation Sciences, University of Queensland, Brisbane, Australia

[13] Intensive Care Medicine Nepean Hospital, New South Wales, Australia

[14] Fiona Stanley Hospital, Perth, Western Australia, Australia

[15] Physiotherapy Department, Princess Alexandra Hospital, Metro South Health, Queensland, Australia

[16] Physiotherapy, St Vincent's Hospital, Sydney, New South Wales, Australia

[17] Department of Anaesthesiology and Perioperative Medicine, Central Clinical School, Monash University, Melbourne, Victoria, Australia

[18] Faculty of Medicine, Nursing and Midwifery, Health Sciences and Physiotherapy, The University of Notre Dame Australia, Perth, Australia

[19] Department of Physiotherapy, Monash Health, Melbourne, Victoria, Australia

[20] Physiotherapy Department, St Vincent’s Hospital, Melbourne, Victoria, Australia

[21]Canberra Health Services, Canberra, Australia

[22] Medical School, Australia National University, Canberra, Australia

[23] Department of Physiotherapy, Division of Allied Health, Austin Health, Melbourne, Australia

[24]Department of Physiotherapy, The University of Melbourne, Melbourne, Australia

[25] Department of Physiotherapy, Royal Brisbane and Women's Hospital, Brisbane, Australia

[26] Data Analytics Research and Evaluation (DARE) Centre, Austin Hospital, Melbourne, Australia

[27] Department of Critical Care Medicine, Hospital Israelita Albert Einstein, Sao Paulo, Brazil

Corresponding author:

Professor Carol Hodgson

Australian and New Zealand Intensive Care Research Centre, Monash University

553 St Kilda Rd, Melbourne, Australia 3004

Email: carol.hodgson@monash.edu

Table of Contents

ADDITIONAL METHODS **5**

eTable 1 - Sites Participating in the Study **10**

eTable 2 - Measurement Tools Used at 6-Month Follow-Up **11**

eTable 3 - Baseline Characteristics and Clinical Outcomes of the Included Patients According to 6-Month Outcome **12**

eTable 4 - Interventions and Development of Complications in the Included Patients **14**

eTable 5 - Interventions and Development of Complications in the Included Patients According to 6-Month Outcome **15**

eTable 7 - Univariable Analyses of Factors Associated with Development of New Disability or Death at 6 Months **18**

eFigure 1 - Roadmap of Restrictions in Australia During the 2020 COVID-19 Pandemic **20**

eFigure 2 - Study Flowchart........Error! Bookmark not defined.

eFigure 3 - Persistent Symptoms at 6 Months........Error! Bookmark not defined.

ADDITIONAL METHODS

Roadmap of restrictions in Australia

The following restrictions were applied in Australia (available in https://www1.health.gov.au/internet/main/publishing.nsf/Content/novel_coronavirus_2019_ncov_weekly_epidemiology_reports_australia_2020.htm):

1. **25 January 2020**: First confirmed COVID-19 case reported in Australia;
2. **01 February 2020**: Travel restrictions on foreign travelers coming from mainland China implemented;
3. **05 February 2020**: Limited local transmission within a cluster first reported in Australia;
4. **01 March 2020**: First COVID-19 death reported in Australia and travel restrictions on foreign travelers coming from Islamic Republic of Iran implemented;
5. **05 March 2020**: Restrictions on travelers from Republic of Korea;
6. **08 March 2020**: Recommended restrictions on COVID-19 contacts and travelers from listed higher risk countries;
7. **11 March 2020**: Restrictions on travelers from Italy;
8. **15 March 2020**: All overseas arrivals required to self-quarantine for 14 days and cruise ship arrivals banned;
9. **16 March 2020**: Non-essential static gatherings of >500 people banned;
10. **18 March 2020**: Restrictions on indoor gatherings;
11. **20 March 2020**: Travel ban on foreign nationals entering Australia and restriction on travel to remote communities (borders closed);
12. **21 March 2020**: Select states and territories close borders to non-essential ravel;

13. **23 March 2020**: Scale up of social distancing measures (stage 1 lockdown);
14. **25 March 2020**: Scale up of social distancing measures (stage 2 lockdown);
15. **28 March 2020**: All people entering Australia required to undertake a mandatory 14-day quarantine at designated facilities (e.g., hotels) in their port of arrival;
16. **29 March 2020**: Public gatherings limited to two persons (stage 3 lockdown);
17. **27 April 2020**: Start of easing restrictions in selected states and territories;
18. **08 May 2020**: Government announced three-step plan to ease COVID-19 restrictions (implementation to vary in states and territories);
19. **05 June 2020**: Western Australia and Northern Territory commenced step 3 easing of restrictions;
20. **01 July 2020**: Victoria implements lockdowns on 'hotspot' suburbs and State of Emergency;
21. **08 July 2020**: New South Wales and Victoria border closes, and stage 3 lockdown of Melbourne and Mitchell Shire;
22. **19 July 2020**: Use of masks is compulsory in Victoria;
23. **02 August 2020**: Stage 4 restrictions for metropolitan Melbourne and stage 3 restrictions for regional Victoria and State of Disaster declared in Victoria;
24. **13 September 2020**: Victoria commenced easing of restrictions against the 'roadmap to reopening';

25. **16 October 2020**: Select states and territories commence opening for quarantine free travel from New Zealand; and
26. **18 October 2020**: Further easing of restrictions in Victoria.

Definition of stage lockdowns

Stage 3 lockdown

1. Stay at home order (leave only for shopping food or essential items, medical care and caregiving, work and study [if you can’t do from home] and exercise [but don’t leave or enter restricted areas);
2. No private visitors and public gathering of up to 2 people only;
3. Work from home;
4. Remote learning;
5. Community sport, indoor sport, swimming pools, playcenters and playgrounds closed;
6. Restaurants, bars and pubs for take away and delivery only;
7. Saunas and personal care services closed;
8. Weddings up to 5 people and funerals up to 10 people;
9. Galleries, cinemas, concert venues, libraries, museums, casinos and stadiums closed.

Stage 4 lockdown

In addition to the points described above for the stage 3 lockdown:

1. Curfew from 8PM to 5AM;
2. Night Network suspended and public transport services reduced during curfew hours;

3. Outdoor exercise limited to 1 hour per day and no more than 5 kilometers from your home;
4. Shopping limited to one person per household per day and limited to 5 kilometers;
5. Reduction in workforce of industries, markets and companies.

Treatment and outcomes definition

An antiviral agent refers to any agent(s) prescribed to treat or prevent viral infections by interfering with the viral replication cycle. Examples of antivirals include neuraminidase inhibitors (e.g., oseltamivir), ribavirin, acyclovir, remdesevir and lopinavir/ritonavir (note that other examples exist). Topical preparations are not included.

Viral pneumonitis was defined as pneumonia (pneumonitis) that is believed to occur as a direct consequence of an infecting virus/infecting viruses. Viral pneumonitis may be a clinical diagnosis, with or without radiographic or histopathological evidence of lung consolidation. Although preferred, identification of the infecting viral species is not essential to make the diagnosis. Bacterial pneumonia was defined as pneumonia (pneumonitis) that is believed to occur as a direct consequence of infecting bacteria. It is an acute infection of the lung parenchyma caused by bacteria (e.g., Streptococcus pneumoniae, Haemophilus influenzae, Chlamydia pneumoniae, Mycoplasma pneumoniae, and Legionella pneumophila). Signs and symptoms include productive cough, fever, chills, shortness of breath, and chest pain. Bacterial pneumonia may be a clinical diagnosis, with or without radiographic or histopathological evidence of

lung consolidation. Although preferred, identification of the infecting bacterial species is not essential to make the diagnosis.

eTable 1 - Sites Participating in the Study

	Number of Patients
Alfred Hospital ICU	19
Angliss Hospital	1
Austin Hospital ICU	17
Box Hill Hospital ICU	2
Cabrini Hospital ICU	5
Canberra Hospital ICU	1
Casey Hospital ICU	4
Concord Hospital (Sydney) ICU	2
Dandenong Hospital ICU	12
Epworth Hospital (Richmond) ICU	5
Footscray Hospital ICU	18
Frankston Hospital ICU	2
Gold Coast University Hospital ICU	2
John Hunter Hospital ICU	6
Launceston General Hospital ICU	1
Monash Medical Centre	6
Nepean Hospital ICU	6
Princess Alexandra Hospital ICU	3
Redcliffe Hospital ICU	1
Royal Adelaide Hospital ICU	4
Royal Brisbane and Women's Hospital ICU	3
Royal Melbourne Hospital ICU	43
Royal North Shore Hospital ICU	9
Royal Prince Alfred Hospital ICU	44
St George Hospital (Sydney) ICU	6
St Vincent's Hospital (Melbourne) ICU	19
St Vincent's Hospital (Sydney) ICU	1
Sunshine Hospital ICU	24
The Prince Charles Hospital ICU	1
Westmead Hospital ICU	7
**Total**	**274**

eTable 2 - Measurement Tools Used at 6-Month Follow-Up

Tool	Description	Domain	Scoring
WHODAS 2.0 12L	Global health and disability	Disability	Total score 0-48, converted to a percentage (0-100%)<br>Disability: WHODAS ≥ 25%<br>New disability: increase in 10% from baseline WHODAS<br>Categories: no (< 5%), mild (5-24%), moderate (25-49%), severe (50-95%) and complete (≥ 96%)
Return to work	Did not return to work due to health	WHODAS - work	Yes / No
EQ5D-5L	Health status	HRQoL	Utility score -0.6 to +1 (UK crosswalk)<br>Visual analogue score 0-100<br>Domains: No problem = 1; Extreme problem = 5<br>New problems: Score of the specific component at 6 months was higher than at baseline.
HADS	Subjective report of anxiety and depression	Psychological function	Total score of 0 (none) to 21 (severe) for each of the two subscales. Subscale scores ≥ 8 indicate clinically significant anxiety or depression
IES-6	Subjective report of stress caused by traumatic events	Psychological function	6 questions screening for PTSD<br>Mean scores ≥ 1.75 indicated high risk of PTSD
MoCA-Blind	Montreal Cognitive Assessment to screen cognition	Cognitive function	Includes memory, attention, language, abstraction, delayed recall and orientation.<br>Total score of 22<br>MoCA-Blind < 18 indicate impaired cognition
IADL	Instrumental activities of daily living	Activities of daily living	Score 0-8<br>Fully independent = 8

Abbreviations: WHODAS: The World Health Organization Disability Assessment Schedule 2.0 12 level; HADS: Hospital Anxiety and Depression Scale; IES-6: Impact of Events Scale – 6 questions; MoCA-Blind: Montreal Cognitive Assessment Score – Blind; IADL: Instrumental activities of Daily Living;

eTable 3 - Baseline Characteristics and Clinical Outcomes of the Included Patients According to 6-Month Outcome

	Responders[a] (*n* = 115)	Non-Responders (*n* = 54)	Deceased (*n* = 43)	*p* value[b]	*p* value[c]
Age, years	58 (51 - 69)	53 (40 - 66)	72 (64 - 75.6)	0.067	< 0.001
< 60	62 (53.9)	32 (59.3)	5 (11.6)	0.738	< 0.001
60 - 69	28 (24.3)	11 (20.4)	15 (34.9)		
70 - 79	23 (20.0)	9 (16.7)	16 (37.2)		
> 80	2 (1.7)	2 (3.7)	7 (16.3)		
Male gender - no. (%)	66 (57.4)	29 (53.7)	29 (67.4)	0.740	0.386
APACHE II	13.0 (10.0 - 16.8)	12 (8 - 17)	19.0 (17.0 - 23.5)	0.702	< 0.001
Days between symptoms - hospital admission	7.1 (4.3 - 9.4)	5.5 (4.2 - 9.2)	3.9 (2.4 - 7.3)	0.290	0.004
Days between symptoms - ICU admission	8.6 (6.1 - 11.1)	7.6 (5.3 - 11.0)	6.6 (4.1 - 9.7)	0.323	0.031
Body mass index, kg/m^2	29.9 (25.7 - 35.9)	27.9 (24.4 - 34.6)	30.2 (25.0 - 34.6)	0.182	0.399
Clinical frailty score	2 (2 - 3)	3 (2 - 3)	3 (3 - 4)	0.789	< 0.001
Years of education	14 (11 - 16)	---	---	---	---
Healthcare worker - no. (%)	14 (13.0)	9 (16.7)	1 (2.3)	0.634	0.055
Co-existing disorders - no. (%)					
Diabetes	35 (32.1)	12 (22.2)	20 (46.5)	0.205	0.044
Obesity	34 (31.8)	13 (25.5)	13 (31.0)	0.461	0.733
Use of ACEi or ARB	19 (18.3)	7 (13.5)	12 (28.6)	0.503	0.169
Chronic cardiac failure	12 (11.1)	6 (11.5)	17 (40.5)	0.999	< 0.001
Smoker	10 (9.5)	7 (13.7)	8 (19.5)	0.425	0.255
Chronic pulmonary disease**	5 (4.6)	2 (4.0)	9 (20.9)	0.999	0.005
Asthma	13 (12.0)	10 (19.6)	5 (11.6)	0.231	0.412
Immunosuppression	12 (11.1)	4 (7.7)	8 (19.0)	0.585	0.235
Chronic kidney disease	5 (4.6)	2 (3.8)	7 (16.3)	0.999	0.046
Chronic hematological disease	3 (2.8)	1 (1.9)	5 (11.9)	0.999	0.039
Cancer	4 (3.7)	1 (1.9)	7 (16.3)	0.999	0.008
Symptoms - no. (%)					
Fever	86 (80.4)	41 (75.9)	32 (74.4)	0.543	0.657
Cough	77 (72.0)	35 (64.8)	31 (72.1)	0.369	0.623
Shortness of breath	72 (67.3)	36 (66.7)	27 (62.8)	0.999	0.869
Fatigue	57 (53.3)	27 (50.0)	22 (51.2)	0.740	0.930
Myalgia	51 (47.7)	10 (18.5)	12 (27.9)	< 0.001	0.001
Diarrhea	29 (27.1)	17 (31.5)	13 (30.2)	0.583	0.786
Sore throat	27 (25.2)	11 (20.4)	8 (18.6)	0.559	0.662
Anosmia	12 (11.2)	6 (11.1)	4 (9.3)	0.999	0.999
Runny nose	12 (11.2)	5 (9.3)	5 (11.6)	0.792	0.912
Signs at baseline					
Heart rate, bpm	97 (85 - 109)	105 (90 - 118)	100 (88 - 113)	0.038	0.100
Respiratory rate, breaths/min	30 (24 - 38)	30 (24 - 40)	28 (21 - 38)	0.681	0.628
Mean arterial pressure, mmHg	80 (69 - 97)	76 (69 - 94)	84 (71 - 97)	0.490	0.488
Temperature, ºC	38.2 (37.5 - 38.9)	38.4 (37.3 - 39.1)	38.0 (37.4 - 38.8)	0.335	0.446
SpO_2, %	92 (88 - 95)	92 (89 - 95)	92 (84 - 95)	0.502	0.388
Clinical outcomes					

eTable 3 - Baseline Characteristics and Clinical Outcomes of the Included Patients According to 6-Month Outcome

	Responders[a] (*n* = 115)	Non-Responders (*n* = 54)	Deceased (*n* = 43)	*p* value[b]	*p* value[c]
Hospital readmission - no. (%)	18 (17.0)	0 (0.0)	---	0.999	---
Duration of ventilation, days	12.0 (5.0 - 16.0)	8.0 (5.5 - 16.5)	16.0 (6.5 - 20.5)	0.905	0.329
ICU length of stay, days	6.5 (3.1 - 16.1)	4.7 (2.6 - 10.8)	12.9 (6.0 - 21.7)	0.227	0.005
Hospital length of stay, days	17.2 (10.1 - 33.0)	12.9 (8.2 - 23.0)	16.1 (9.9 - 24.2)	0.125	0.257
ICU mortality - no. (%)	0 (0.0)	0 (0.0)	39 (90.7)	---	< 0.001
Hospital mortality - no. (%)	0 (0.0)	0 (0.0)	42 (97.7)	---	< 0.001

Data are median (quartile 25% - quartile 75%) or No (%). Percentages may not total 100 because of rounding.

Abbreviations: APACHE: Acute Physiology and Chronic Health Evaluation; ACEi: angiotensin converting enzyme inhibitor; ARB: angiotensin II receptor blocker; ICU: intensive care unit.

[a] The follow-up cohort comprises patients who were contacted successfully at 6 months. However, data may be missing for some outcomes depending on the willingness of the patient to answer all questions.

[b] *p* value for the comparison of responders vs. non-responders.

[c] *p* value for the comparison of the three groups.

* On a scale from 1 to 10 (1 would be the lowest level of financial distress).

** Not considering asthma.

eTable 4 - Interventions and Development of Complications in the Included Patients

	Hospital Cohort (*n* = 212)	Follow-Up Cohort[a] (*n* = 160)
Scales		
MRC MMT-SS at ICU discharge	48 (34 - 60)	48 (29 - 60)
ICU acquired weakness - no. (%)	58 / 137 (42.3)	51 (49.5)
Highest ICU mobility scale while in ICU	5 (3 - 8)	5 (2 - 8)
Interventions - no. (%)		
Drugs		
Antibiotics	188 / 205 (91.7)	140 / 153 (91.5)
Bacteria identified during hospitalization	45 / 193 (23.3)	33 /143 (23.1)
Steroids	147 / 205 (71.7)	108 / 153 (70.6)
Hydroxychloroquine	15 / 205 (7.3)	13 / 153 (8.5)
Oseltamivir	0 / 205 (0.0)	0 / 153 (0.0)
Lopinavir-Ritonavir	7 / 205 (3.4)	7 / 153 (4.6)
Remdesevir	64 / 212 (30.2)	44 / 160 (27.5)
Organ support*		
High-flow nasal cannula	129 / 206 (62.6)	93 / 155 (60.0)
Non-invasive ventilation	29 / 203 (14.3)	21 / 152 (13.8)
Mechanical ventilation	120 / 210 (57.1)	98 / 159 (61.6)
Inotropic or vasopressor	112 / 203 (55.2)	91 / 152 (59.9)
Neuromuscular blocking agent	91 / 203 (44.8)	76 / 152 (50.0)
Prone positioning	78 / 203 (38.4)	61 / 152 (40.1)
Renal replacement therapy	28 / 203 (13.8)	25 / 152 (16.4)
Other cardiac procedures	13 / 203 (6.4)	13 / 152 (8.6)
Tracheostomy	21 / 203 (10.3)	15 / 152 (9.9)
Inhaled nitric oxide	13 / 203 (6.4)	13 / 152 (8.6)
Extracorporeal membrane oxygenation	7 / 203 (3.4)	7 / 152 (4.6)
Development of complications - no. (%)		
Viral pneumonitis	128 / 203 (63.1)	95 / 151 (62.9)
Bacterial pneumonia	47 / 196 (24.0)	35 / 146 (24.0)
Bacteremia	22 / 199 (11.1)	19 / 148 (12.8)
Stroke	1 / 204 (0.5)	1 / 153 (0.7)
Arrhythmia	30 / 204 (14.7)	23 / 153 (15.0)
Barotrauma**	10 / 204 (4.9)	9 / 153 (5.9)
Cardiac arrest	2 / 204 (1.0)	2 / 153 (1.3)
Pulmonary embolism	9 / 197 (4.6)	6 / 147 (4.1)
Deep vein thrombosis	12 / 197 (6.1)	11 / 147 (7.5)
Myocarditis	19 / 196 (9.7)	16 / 147 (10.9)

Data are median (quartile 25% - quartile 75%) or No (%). Percentages may not total 100 because of rounding.

[a] The follow-up cohort comprises patients who died within 6 months or who were contacted successfully at 6 months. However, data may be missing for some outcomes depending on the willingness of the patient to answer all questions.

* Assessed daily until ICU discharge

** Defined as pneumothorax or pneumomediastinum or subcutaneous emphysema on chest X-ray or CT chest

eTable 5 - Interventions and Development of Complications in the Included Patients According to 6-Month Outcome

	Responders[s] (*n* = 115)	Non-Responders (*n* = 54)	Deceased (*n* = 43)	*p* value[b]	*p* value[c]
Scales					
MRC MMT-SS at ICU discharge	49 (42 - 60)	60 (48 - 60)	0 (0 - 8)	0.166	< 0.001
ICU acquired weakness - no. (%)	29 / 76 (38.2)	9 / 36 (25.0)	20 / 25 (80.0)	0.203	< 0.001
Highest ICU mobility scale while in ICU	6 (5 - 8)	6 (3 - 9)	0 (0 - 2)	0.420	< 0.001
Interventions - no. (%)					
Drugs					
Antibiotics	96 (88.9)	50 (92.6)	42 (97.7)	0.582	0.212
Bacteria identified during hospitalization	18 (18.4)	13 (25.0)	14 (32.6)	0.495	0.171
Steroids	73 (67.6)	40 (74.1)	34 (79.1)	0.470	0.359
Hydroxychloroquine	7 (6.5)	2 (3.7)	6 (14.0)	0.719	0.163
Oseltamivir	0 (0.0)	0 (0.0)	0 (0.0)	---	---
Lopinavir-Ritonavir	5 (4.6)	0 (0.0)	2 (4.7)	0.170	0.230
Remdesevir	36 (31.3)	21 (38.9)	7 (16.3)	0.384	0.047
Organ support*					
High-flow nasal cannula	67 (60.9)	37 (69.8)	25 (58.1)	0.300	0.435
Non-invasive ventilation	12 (11.2)	8 (15.1)	9 (20.9)	0.612	0.294
Mechanical ventilation	57 (50.0)	24 (45.3)	39 (90.7)	0.620	< 0.001
Inotropic or vasopressor	52 (48.6)	22 (41.5)	38 (88.4)	0.406	< 0.001
Neuromuscular blocking agent	40 (37.4)	16 (30.2)	35 (81.4)	0.386	< 0.001
Prone positioning	38 (35.5)	18 (34.0)	22 (51.2)	0.999	0.157
Renal replacement therapy	12 (11.2)	3 (5.7)	13 (30.2)	0.389	0.003
Other cardiac procedures	8 (7.5)	0 (0.0)	5 (11.6)	0.053	0.029
Tracheostomy	9 (8.4)	7 (13.2)	5 (11.6)	0.404	0.557
Inhaled nitric oxide	7 (6.5)	0 (0.0)	6 (14.0)	0.096	0.015
Extracorporeal membrane oxygenation	4 (3.7)	0 (0.0)	3 (7.0)	0.303	0.135
Development of complications - no. (%)					
Viral pneumonitis	66 (62.3)	34 (63.0)	28 (65.1)	0.999	0.981
Bacterial pneumonia	19 (18.4)	12 (23.1)	16 (39.0)	0.527	0.037
Bacteremia	10 (9.5)	4 (7.5)	8 (19.5)	0.775	0.169
Stroke	0 (0.0)	0 (0.0)	1 (2.3)	---	0.211
Arrhythmia	12 (11.1)	7 (13.2)	11 (25.6)	0.796	0.092
Barotrauma**	5 (4.6)	2 (3.8)	3 (7.0)	0.999	0.758
Cardiac arrest	0 (0.0)	0 (0.0)	2 (4.7)	---	0.044
Pulmonary embolism	4 (3.8)	3 (5.8)	2 (4.9)	0.687	0.902
Deep vein thrombosis	8 (7.7)	1 (1.9)	3 (7.3)	0.273	0.347
Myocarditis	7 (6.7)	3 (5.9)	9 (22.0)	0.999	0.022

Data are median (quartile 25% - quartile 75%) or No (%). Percentages may not total 100 because of rounding.

[a] The follow-up cohort comprises patients who were contacted successfully at 6 months. However, data may be missing for some outcomes depending on the willingness of the patient to answer all questions.

[b] *p* value for the comparison of responders vs. non-responders.

[c] *p* value for the comparison of the three groups.

* Assessed daily until ICU discharge

** Defined as pneumothorax or pneumomediastinum or subcutaneous emphysema on chest X-ray or CT chest

eTable 6 - WHODAS Components at 6 Months in Patients Included

	Follow-Up Cohort[a] (*n* = 115)	New Disability Yes (*n* = 42)	New Disability No (*n* = 66)	*p* value
Standing for long periods such as 30 minutes				< 0.001
None	68 (60.2)	15 (35.7)	51 (77.3)	
Mild	17 (15.0)	8 (19.0)	7 (10.6)	
Moderate	17 (15.0)	12 (28.6)	5 (7.6)	
Severe	4 (3.5)	3 (7.1)	1 (1.5)	
Extreme or cannot do	7 (6.2)	4 (9.5)	2 (3.0)	
Taking care of your household responsibilities				< 0.001
None	77 (68.1)	13 (31.0)	61 (92.4)	
Mild	14 (12.4)	9 (21.4)	4 (6.1)	
Moderate	17 (15.0)	16 (38.1)	1 (1.5)	
Severe	3 (2.7)	3 (7.1)	0 (0.0)	
Extreme or cannot do	2 (1.8)	1 (2.4)	0 (0.0)	
Learning a new task				0.001
None	94 (83.2)	28 (66.7)	62 (93.9)	
Mild	10 (8.8)	7 (16.7)	3 (4.5)	
Moderate	8 (7.1)	6 (14.3)	1 (1.5)	
Severe	1 (0.9)	1 (2.4)	0 (0.0)	
Joining in community activities in the same way as anyone else can?				< 0.001
None	81 (71.7)	16 (38.1)	61 (92.4)	
Mild	12 (10.6)	11 (26.2)	1 (1.5)	
Moderate	11 (9.7)	8 (19.0)	3 (4.5)	
Severe	4 (3.5)	4 (9.5)	0 (0.0)	
Extreme or cannot do	5 (4.4)	3 (7.1)	1 (1.5)	
Emotionally affected by your health problems				< 0.001
None	38 (33.6)	5 (11.9)	32 (48.5)	
Mild	31 (27.4)	10 (23.8)	20 (30.3)	
Moderate	27 (23.9)	15 (35.7)	11 (16.7)	
Severe	13 (11.5)	10 (23.8)	2 (3.0)	
Extreme or cannot do	4 (3.5)	2 (4.8)	1 (1.5)	
Concentrating on doing something for ten minutes				0.015
None	83 (73.5)	25 (59.5)	55 (83.3)	
Mild	17 (15.0)	9 (21.4)	8 (12.1)	
Moderate	9 (8.0)	5 (11.9)	3 (4.5)	
Severe	4 (3.5)	3 (7.1)	0 (0.0)	
Walking a long distance such as a kilometer				< 0.001
None	57 (50.4)	9 (21.4)	45 (68.2)	
Mild	15 (13.3)	7 (16.7)	8 (12.1)	
Moderate	14 (12.4)	7 (16.7)	6 (9.1)	
Severe	10 (8.8)	8 (19.0)	2 (3.0)	
Extreme or cannot do	17 (15.0)	11 (26.2)	5 (7.6)	

eTable 6 - WHODAS Components at 6 Months in Patients Included

	Follow-Up Cohort[a] (*n* = 115)	New Disability: Yes (*n* = 42)	New Disability: No (*n* = 66)	*p* value
Washing your whole body				< 0.001
None	99 (88.4)	31 (73.8)	65 (98.5)	
Mild	6 (5.4)	5 (11.9)	1 (1.5)	
Moderate	3 (2.7)	3 (7.1)	0 (0.0)	
Severe	3 (2.7)	3 (7.1)	0 (0.0)	
Extreme or cannot do	1 (0.9)	0 (0.0)	0 (0.0)	
Getting dressed				0.001
None	94 (83.9)	29 (69.0)	62 (93.9)	
Mild	12 (10.7)	9 (21.4)	3 (4.5)	
Moderate	3 (2.7)	3 (7.1)	0 (0.0)	
Severe	1 (0.9)	1 (2.4)	0 (0.0)	
Extreme or cannot do	2 (1.8)	0 (0.0)	1 (1.5)	
Dealing with people you do not know				0.001
None	96 (85.0)	29 (69.0)	63 (95.5)	
Mild	7 (6.2)	5 (11.9)	2 (3.0)	
Moderate	5 (4.4)	4 (9.5)	1 (1.5)	
Severe	3 (2.7)	3 (7.1)	0 (0.0)	
Extreme or cannot do	2 (1.8)	1 (2.4)	0 (0.0)	
Maintaining a friendship				0.003
None	102 (91.1)	34 (81.0)	65 (98.5)	
Mild	4 (3.6)	3 (7.1)	1 (1.5)	
Moderate	2 (1.8)	2 (4.8)	0 (0.0)	
Severe	3 (2.7)	3 (7.1)	0 (0.0)	
Extreme or cannot do	1 (0.9)	0 (0.0)	0 (0.0)	
Your day-to-day work/school				< 0.001
None	70 (61.9)	8 (19.0)	58 (87.9)	
Mild	15 (13.3)	12 (28.6)	3 (4.5)	
Moderate	12 (10.6)	8 (19.0)	4 (6.1)	
Severe	4 (3.5)	4 (9.5)	0 (0.0)	
Extreme or cannot do	12 (10.6)	10 (23.8)	1 (1.5)	

[a] The follow-up cohort comprises patients who were contacted successfully at 6 months. However, data may be missing for some outcomes depending on the willingness of the patient to answer all questions.

eTable 7 - Analyses of Factors Associated with Development of New Disability or Death at 6 Months

	Univariable analyses		Full multivariable analysis*		Final model**	
	Odds Ratio (95% CI)	*p* value	Odds Ratio (95% CI)	*p* value	Odds Ratio (95% CI)	*p* value
Age	1.04 (1.01 to 1.07)	0.010	1.08 (0.73 to 1.60)	0.705	---	---
Male gender	0.79 (0.40 to 1.58)	0.509	---	---	---	---
APACHE II	1.11 (1.05 to 1.18)	0.001	1.21 (0.76 to 1.92)	0.429	1.08 (1.01 to 1.17)	0.032
Body mass index	1.00 (0.97 to 1.03)	0.937	---	---	---	---
Clinical frailty score	1.70 (1.23 to 2.35)	0.001	1.52 (0.98 to 2.38)	0.063	1.49 (1.05 to 2.11)	0.025
Years of education	0.98 (0.90 to 1.07)	0.649	---	---	---	---
Diabetes	2.63 (1.27 to 5.47)	0.009	1.96 (0.84 to 4.56)	0.121	---	---
Obesity	1.60 (0.74 to 3.47)	0.232	---	---	---	---
Use of ACEi or ARB	1.55 (0.64 to 3.74)	0.326	---	---	---	---
Chronic cardiac failure	2.66 (1.05 to 6.73)	0.040	1.29 (0.45 to 3.73)	0.636	---	---
Smoker	1.14 (0.41 to 3.19)	0.806	---	---	---	---
Asthma	1.91 (0.62 to 5.83)	0.259	---	---	---	---
Immunosuppression	1.34 (0.48 to 3.68)	0.576	---	---	---	---
Chronic kidney disease	2.32 (0.59 to 9.09)	0.229	---	---	---	---
Chronic hematological disease	1.31 (0.29 to 5.98)	0.727	---	---	---	---
Cancer	8.24 (1.02 to 66.80)	0.048	5.24 (0.55 to 50.00)	0.150	---	---
Corticosteroids	1.93 (0.93 to 4.01)	0.078	---	---	---	---
Use of invasive ventilation	1.93 (0.95 to 3.91)	0.069	---	---	---	---
Use of inotropes or vasopressors	1.88 (0.95 to 3.71)	0.069	---	---	---	---
Use of neuromuscular blocking agents	2.83 (1.42 to 5.60)	0.003	1.94 (0.81 to 4.60)	0.135	---	---
Prone positioning	2.22 (1.09 to 4.51)	0.028	1.67 (0.71 to 3.93)	0.239	---	---
Use of renal replacement therapy	2.19 (0.82 to 5.82)	0.118	---	---	---	---
Tracheostomy	1.96 (0.57 to 6.72)	0.284	---	---	---	---

eTable 7 - Analyses of Factors Associated with Development of New Disability or Death at 6 Months

	Univariable analyses		Full multivariable analysis*		Final model**	
	Odds Ratio (95% CI)	*p* value	Odds Ratio (95% CI)	*p* value	Odds Ratio (95% CI)	*p* value
Baseline disability	0.24 (0.03 to 2.10)	0.199	---	---	---	---
Baseline WHODAS	0.98 (0.93 to 1.02)	0.338	---	---	---	---
Week of admission	1.01 (0.97 to 1.05)	0.584	---	---	---	---

Abbreviations: APACHE: Acute Physiology and Chronic Health Evaluation; ACEi: angiotensin converting enzyme inhibitor; ARB: angiotensin II receptor blocker; NIV: non-invasive ventilation; HFNC: high-flow nasal cannula; ECMO: extracorporeal membrane oxygenation.

* Full multivariable model forcing all variable with $p < 0.05$ in the univariable model.

** The final model was constructed using a least absolute shrinkage and selection operator approach and confirmed using a backwards elimination technique before undergoing a final assessment for clinical and biological plausibility.

eFigure 1 - Roadmap of Restrictions in Australia During the 2020 COVID-19 Pandemic

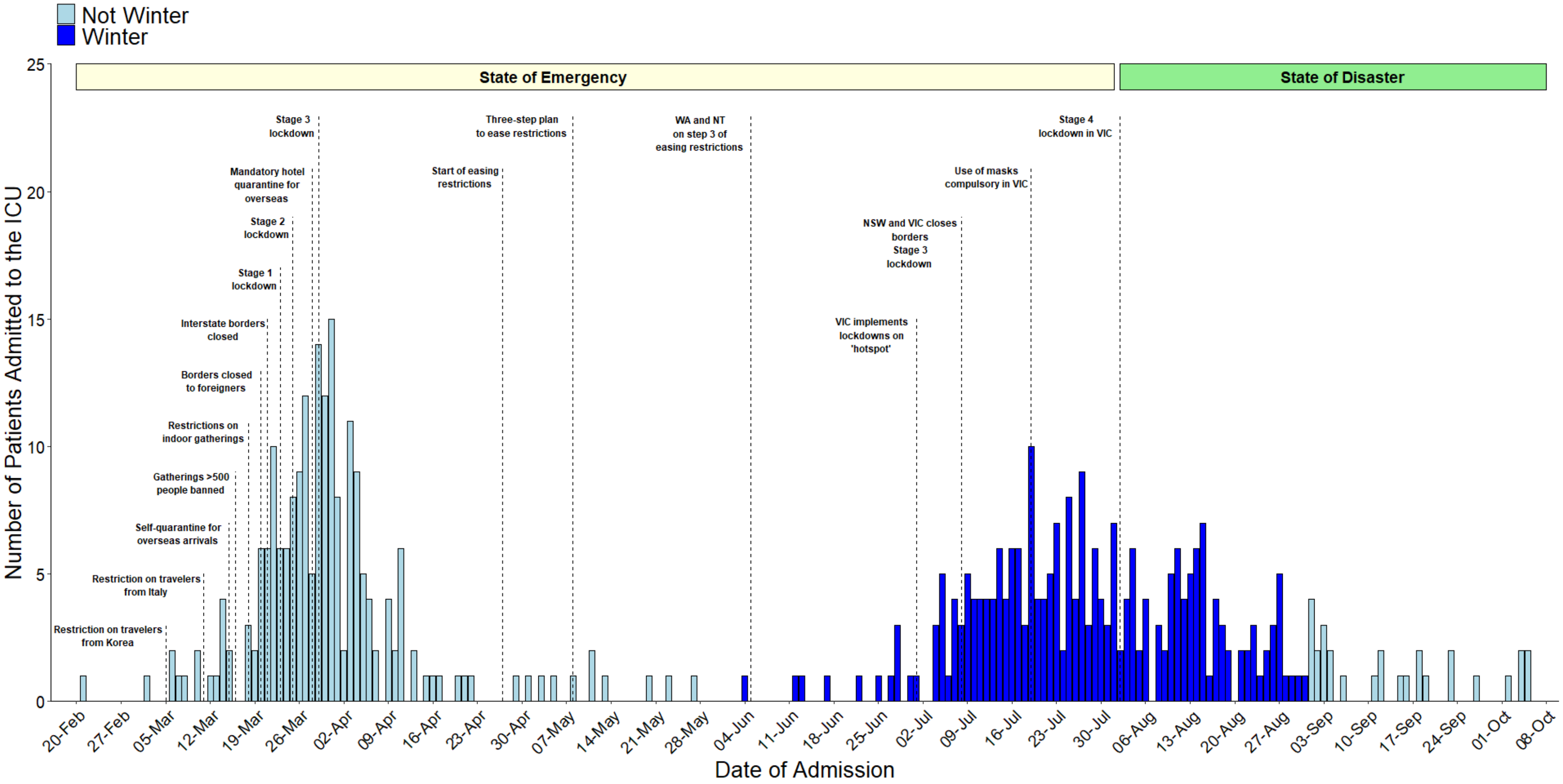

More information and definition of the stage lockdowns are available in the Additional Methods section. WA denotes Western Australia, NT Northern Territory, VIC Victoria and NSW New South Wales.

eFigure 2. Kaplan-Meier curve of survival to day 180, including patients who received mechanical ventilation (red) and patients who were not mechanically ventilated (blue).

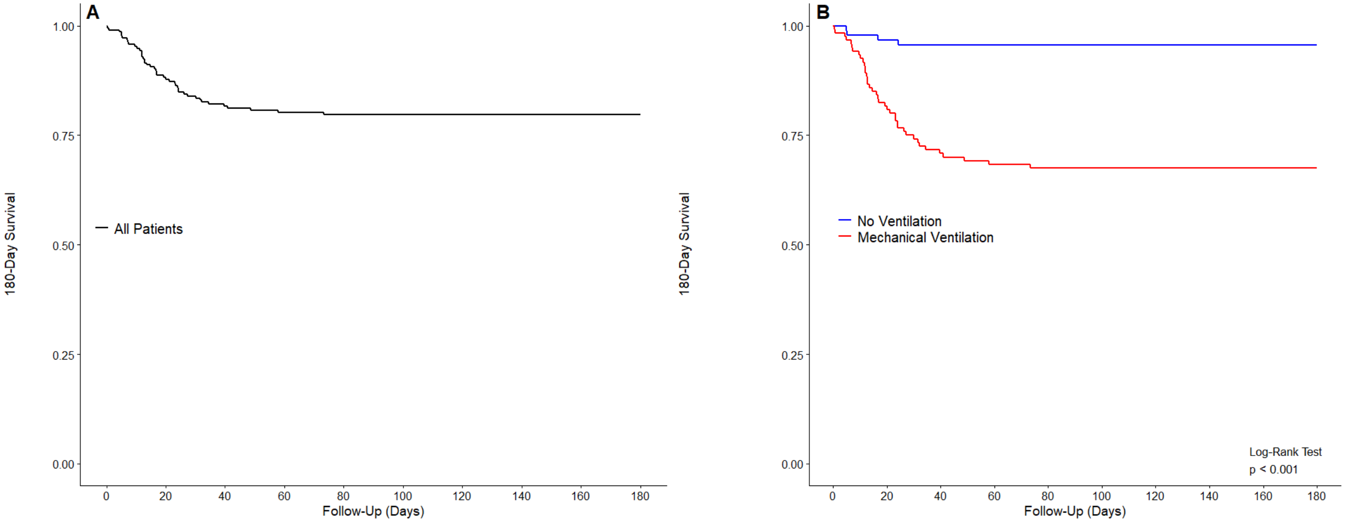

No. at Risk

	0	20	40	60	80	100	120	140	160	180
All Patients	212	187	173	170	169	169	169	169	169	169

No. at Risk

	0	20	40	60	80	100	120	140	160	180
No Ventilation	90	87	86	86	86	86	86	86	86	86
Mechanical Ventilation	120	98	85	82	81	81	81	81	81	81
